# Unintended Consequences of Conservation Actions: Managing Disease in Complex Ecosystems

**DOI:** 10.1371/journal.pone.0028671

**Published:** 2011-12-07

**Authors:** Aliénor L. M. Chauvenet, Sarah M. Durant, Ray Hilborn, Nathalie Pettorelli

**Affiliations:** 1 Institute of Zoology, Zoological Society of London, London, United Kingdom; 2 Division of Biology, Imperial College London, Ascot, United Kingdom; 3 Tanzania Wildlife Research Institute, Arusha, Tanzania; 4 Wildlife Conservation Society, Bronx, New York, United States of America; 5 School of Aquatic and Fishery Sciences, University of Washington, Seattle, United States of America; University of Western Ontario, Canada

## Abstract

Infectious diseases are increasingly recognised to be a major threat to biodiversity. Disease management tools such as control of animal movements and vaccination can be used to mitigate the impact and spread of diseases in targeted species. They can reduce the risk of epidemics and in turn the risks of population decline and extinction. However, all species are embedded in communities and interactions between species can be complex, hence increasing the chance of survival of one species can have repercussions on the whole community structure. In this study, we use an example from the Serengeti ecosystem in Tanzania to explore how a vaccination campaign against Canine Distemper Virus (CDV) targeted at conserving the African lion (*Panthera leo*), could affect the viability of a coexisting threatened species, the cheetah (*Acinonyx jubatus*). Assuming that CDV plays a role in lion regulation, our results suggest that a vaccination programme, if successful, risks destabilising the simple two-species system considered, as simulations show that vaccination interventions could almost double the probability of extinction of an isolated cheetah population over the next 60 years. This work uses a simple example to illustrate how predictive modelling can be a useful tool in examining the consequence of vaccination interventions on non-target species. It also highlights the importance of carefully considering linkages between human-intervention, species viability and community structure when planning species-based conservation actions.

## Introduction

Whilst diseases are often a natural component of ecosystems [1], the impacts of endemic and emerging diseases on biodiversity in an increasingly human-modified landscape have become a cause for concern [1], [2]. Diseases are known to impact biodiversity by suppressing population growth rate and increasing vulnerability to extinction [3]. They have caused the severe decline of some species (e.g., Ethiopian wolf *Canis simensis*) [4], local extinctions (e.g., African wild dog *Lycaon pictus*) [5] and global extinctions (e.g., several amphibian species) [6]. Disease control can be implemented through the erection of fences, which limit contact with infected individuals [7], culling [8] or vaccination of infected and susceptible animals [4], [9]. These measures can have negative consequences for wild populations. For example, in the Kalahari, fences that were erected to protect livestock between the 1950s and 1990s were partly responsible for the catastrophic decrease of the local wildebeest population (*Connochaetes taurinus*) [7]; the attempt to control the spread of tuberculosis to livestock in the United Kingdom has led to the mass culling of European badgers (*Meles meles*) [8]. Clearly, mechanisms for disease control have major impacts on wildlife, and vaccination is increasingly gaining interest as a potential conservation tool [1]. However, its impact on wildlife communities has seldom been investigated. As interactions within a community are complex [10], [11], non-target species can be put under pressure as an unintended consequence of actions taken for another (Table 1) [12]–[16]. If resources are to be used wisely, it is critical to ensure that conservation actions, in general, and disease management, in particular, do not have major unintended negative consequences, such as increasing the vulnerability of perhaps more threatened species.

**Table 1:** Examples of conservation actions that have had unintended negative impact on non-target species.

Conservation actions	Unintended consequences	Reference
Fencing to reduce human-wildlife conflict	Increased pressure on vegetation e.g., in Africa, moderate to high densities of elephants (*Loxodonta africana*) in fenced areas have a significant negative impact on woody vegetation	[12]
Food supplementation	Introduction of alien species e.g., the introduction of American red squirrels (*Tamiasciurus hudsonicus*) to Newfoundland to supplement the diet of declining pine marten (*Martes americana*) is responsible for the decline of Newfoundland red crossbill (*Loxia curvirostra percna*)	[13]
Invasive alien species control	Mesopredator release e.g., following the culling of cats (*Felis catus*) on Macquarie island in 2001, the number of rabbits (*Oryctolagus cuniculus*) increased and led to changes in vegetation composition throughout the island	[14]
Pest management	Introduction of invasive species e.g., cane toad (*Bufo marinus*), introduced as a biological control to the sugar-cane beetle (*Dermolepida albohirtum*), is toxic to Australian wildlife and extending its range	[15]
Creation of artificial permanent water holes	Negative impact on endemic vegetation e.g., in Tembe Elephant Park, South Africa, elephants' path, resting area and feeding area are driven by the proximity to created water holes. Rare endemic sand forest nearby the new artificial waterholes is under pressure.	[16]

Here we make use of a well-known ecosystem in Africa, the Serengeti, to investigate the potential impacts of disease management on two competing threatened species: the cheetah and the lion. The lions of the Serengeti National Park (SNP) were severely affected by an outbreak of Canine Distemper Virus (CDV) in 1994 [17]. This event led to the loss of a third of the lion population, i.e., approximately 1000 animals, from which it took four years for the population to recover [18]. Lions can become infected with CDV when they come in contact with infected domestic dogs (*Canis lupus familiaris*) [19]. In 1996, ‘project life lion’ was launched as a response to the 1994 CDV epidemic [20]–[22]. It had the ambitious and ostensibly laudable aim to create a vaccination cordon around the Serengeti, promoting local lion and African wild dog survival by vaccinating domestic dogs around the SNP against rabies and CDV [20].

While, in the SNP, CDV has been shown to infect lions, spotted hyenas (*Crocuta crocuta*) and bat-eared foxes (*Otocyon megalotis*) [17], there is no evidence of any population level impact on the cheetah population [23], [24]. In this habitat, cheetahs are known to be at low density mainly due to predation by lions [25]–[28]. They have been found to be barely self-replacing, with a deterministic growth rate λ = 0.997 [27]. Lions can kill adult cheetahs [25], yet they tend to primarily kill newborn cubs that are still in the lair, very often killing the entire litter [29]. Spotted hyenas can also kill newborn cheetah cubs. However, of all the deaths by predation observed, ca. 80% can be attributed to lions [30]. Inter-specific competition for resources often drives interactions between co-existing carnivores: in the SNP, however, cheetahs and lions have a low level of diet overlap [31] and cheetah have been found to avoid hunting grounds where lion density is high [26].

Because the lion and cheetah populations have been studied for 30 years [24], [32], this system presents a unique opportunity to quantitatively investigate the potential impact of conservation-oriented vaccination programmes on a non-target, long-lived, species. Within the system considered, a CDV outbreak (or the removal of such a threat) is expected to impact both lion and cheetah numbers. In particular, lethal CDV outbreaks have the potential to cause a sudden drop in lion numbers, which could allow cheetahs to increase its population size. We thus expect that an effective vaccination campaign could have positive impacts on the lion population size, by reducing or suppressing the chance of a lethal CDV outbreak, and negative impacts on cheetahs.

## Materials and Methods

### Study site and species

The Serengeti is a 30,000-km^2^ ecosystem extending over the border between Tanzania and Kenya and defined by the migration of the wildebeest [33]. There are several conservation administrations within the ecosystem. Here we focus on the main grassland areas of the Serengeti National Park. These grassy plains are located in the south-eastern part of the SNP and adjoining Ngorongoro Conservation Area and cover an area ca. 5000-km^2^ [25], [34]. They are home to a small subset of the cheetah and lion populations that reside in the wider Serengeti ecosystem.

Cheetahs are large carnivores with a life cycle that can be divided into three stages: cubs (up to 12 months old), adolescents (13 to 24 months old) and adults (>2 year of age). Cheetah females are solitary and occupy overlapping home ranges, while males can be solitary, territorial, and/or form coalitions [25]. From two years old, female cheetahs are reproductively active. The maximum number of cubs produced per litter is six [28]. Females can become pregnant before the current litter leaves their side, however, the family will separate before the new cubs are born. If the female loses a litter, she can enter oestrous rapidly [25] and produce a new litter in about 4 months [26]. During the first year, and particularly the first two to three months of their lives, cubs are extremely vulnerable to lions' attack [30].

Lions are large-sized carnivores that are territorial and highly social. They live in prides that are composed of 2 to 9 adult females and 2 to 6 adult males [35]. In addition, the prides contain the females' dependent young. They principally feed on migratory species such as wildebeest and zebra (*Equus burchelli*) and can endure high fluctuations in food availability [36]. Females can start reproducing once they reach four years of age and can live up to 18 years old [37].

In the plains of the SNP, regular surveys provide information on cheetah demographic data such as sex-specific abundance. Consistent data is available since 1991. In addition survival and fecundity rates can be found in published literature [24], [28]. Lion demographic data have been collected since 1966. Survival, fecundity, and abundance estimates (up to 2003), are all available in the published literature [32].

### Modelling

Simple interaction models are widespread in the literature and two common types could be envisaged to model lion-cheetah interactions: (1) a simple two-species model (typically built to describe well-known interactions such as predator-prey or parasitism), or (2) a three-species trophic model (typically built to describe inter-specific competition) [38], [39]. Neither is appropriate for this system. Indeed, a key element of the first type is that predators gain from killing victims by increasing their biomass or reproductive output. In the lion-cheetah relationship, lions do not eat cheetah cubs and therefore, cheetah cub death does not directly influence lion population growth; the reason as to why lions target cheetah cubs is unknown [25]. In addition, as described earlier, inter-specific competition for food plays only a minor role in lion-cheetah interactions, rendering the second type of model inadequate. Finally, as cheetahs display continuous reproduction and lifetime reproductive success of females is highly variable, being partly dependent on the fate of each litter [27], [29], a more flexible and complex model was required.

As a result, we built two separate models: an individual-based population model for the cheetahs (hereafter cheetah IBM) and a matrix population model for the lions (hereafter lion model). We did not consider spatial heterogeneity as a factor influencing lion or cheetah populations as (1) both predators' distributions are highly variable in space and time; (2) information was only available from the plains and their borders (where both the lion and cheetah data used here, are collected) [32], [40]; (3) there is no information available to allow the integration of spatial heterogeneity in our modelling approaches.

#### Lion model

We used a pre-breeding, female-only, age-structured matrix population model (*x* = 18 age classes of one year), with an initial population size of 44 females [37], distributed along age-classes according to [41]. While the cheetah IBM was the most complex population model built for the population to date, the lion model was simpler than previously published models [32]. Although less complex, our lion model was designed to capture the main variability in lion numbers, which dictates the cheetah cub's survival estimate to be considered in our IBM. Since our aim was to model variation in lion numbers and not variation in population structure, a simple model sufficed.

We inferred lion age-specific survival rates and CDV mortality rates from [18] (Tables S1). At each time-step, the “regular” age-specific survival rates (Table S1a) were used if no lethal CDV outbreak occurred. If a lethal CDV outbreak occurred, the lower “CDV” age-specific survival rates (Table S1b) were used. Lions reproduced from 3 years old, until they reach 14 years old [37]. The population's reproductive rates were considered to be normally distributed with mean 0.65 and standard deviation 0.11 [37] (referred later as reproduction distribution). The model was density dependent. Based on values reported in [32], the carrying capacity was set to be 60 from 1966 to 1996, and 80 from 1997 onwards. At each time step, the reproductive rate was randomly selected on the right hand-side of the reproduction distribution (values from mean to maximum) if the population was below carrying capacity. If the population was above carrying capacity, the fecundity at this time-step was selected from the left-hand side of the reproduction distribution (values from minimum to mean). Because density was shown to influence disease transmission rate [32], [41], CDV occurrence was set to be density-dependent. To do so, each time the lion population size reached or surpassed 65, the model was set to compare a random number (from a uniform distribution) to a ‘trigger number’ (which depended on how many outbreaks per 60 years we were modelling; Table S2). If the random number was greater than the target number, an outbreak occurred (Table S1b). The trigger number values were obtained by running the lion model with several potential trigger numbers for 500 iterations, to identify values yielding an average of 2, 4 and 6 outbreaks over 60 years.

#### Cheetah model

**Figure 1:**
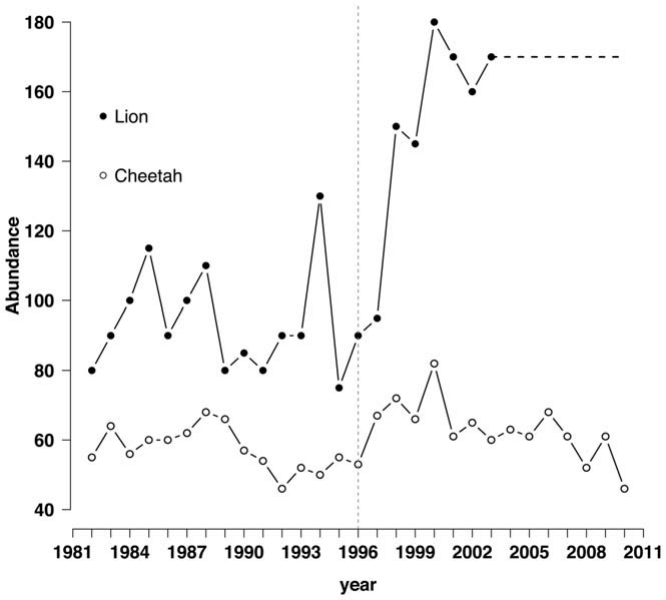
Lion and cheetah abundance in the plains of the Serengeti National Park. Shown is the lion abundance reported in [32] (1982–2003) and the cheetah abundance observed in the field from 1982 to 2010. The horizontal dashed line represents the years for which lion abundance has not been published. The vertical dashed line represents the start of the CDV vaccination campaign.

The cheetah model was individual-based and contained both females and males. The population was structured in three age categories: 0 to 12 months old (cubs), 13 to 24 months old (adolescents) and ≥25 months old (adults). The model followed each individual throughout their life cycle by monthly increments. The initial population number and composition corresponded to year 1991 of the 1991–2010 dataset available for this population (Figure 1; Table S3). Even though cheetah abundance records start in 1982, we chose to focus on data between 1991 and 2010 because this period has consistent observations (i.e., consistent effort and technique used).

The dataset did not contain the number of 0–1 year old cubs as it is impossible to estimate cub abundance given the monitoring method in place [29], [42]. We resolved this by performing a 12-months simulation (500 iterations) for which the initial population contained no 0–1 year old cub. This simulation yielded the average number of 0–1 year old cubs produced during a year: ca. 60. We then used that number as the initial number of 0–1 year old cubs in the population for the full simulations, i.e. run over 60 years. Interestingly, we also tested the model performance, i.e., r-squared, with 10, 30 and 100 initial 0–1 year old cubs in the population and found that the estimated number appeared to have very little influence on performance as a whole.

The monthly survival rates of each age-class were extracted from published literature [24], [28], [29], [42] (Table S4). Depending on the age group they were in, each individual was assigned a probability of survival taken from the normal distribution of Table S4 means and standard deviations. To account for demographic stochasticity, a random number was generated (from a uniform distribution) and compared with the individual assigned survival rate, this at each time-step and for each individual. If the random number was higher than the time-step survival rate, the individual died, if not it lived to the next step. For cubs between 0 and 3 months old, survival also depended on the survival of their littermate. At this age, if a cub died, the entire litter to which it belonged also died [25]. Female cheetah could live up to 15 years and 5 months old while males' longevity was shorter: 11 years and 10 months [24]. Once a female reached adulthood (≥25 months old), it started reproducing (litters contained between 1 and 6 cubs, sex ratio 1∶1). We hypothesised that throughout their lives reproductively active females would always either be with cubs or pregnant. For the first litter, we allowed females to be pregnant before reaching 25 months old (no more than a couple of months) [25]. A new adult female had 1 chance in 4 to give birth to their first litter every month from month 25 of their lives (i.e., randomly assigning pregnancy stages to new adult females). After the first litter was produced, as long as one cub per litter was alive, females did not produce a new litter. If all the cubs died before becoming adults, the females produced a new litter four months after the last cub's death. This allowed three-month gestation and one month to conceive again [42]. If at least one cub reached adulthood, females produced another litter two months later since they could get pregnant before the cubs left. Females could not reproduce past 12 years old [24].

#### Coupling the models

We coupled the IBM with (i) the lions' published abundance [32] and (ii) the lion matrix population model. In order to understand the effect of lion density on cheetah survival, we needed to define the impact of different lion abundances on survival rates of cheetah cubs, which is the key age class affected by lions. However, whilst the mortality of cubs due to lions has been estimated [30], the exact relationship between cub mortality and lion abundance is unknown. To account for such quantitative uncertainty, the abundance of female lions was classified, at each time step, in either of 3 categories: low (≤50 individuals), average (51–79 individuals) and high lion numbers (≥80 individuals). Lion abundance was calculated on a yearly basis. We assumed that lion abundance was constant over a given year, therefore converting a yearly value into twelve identical monthly values. At each time-step of the cheetah model, cubs' survival was influenced by which density category the lions were in (low, average or high). During low lion abundance, cubs' survival was the highest: survival was sampled on the right hand side of the survival distribution (largest 10% on the normal distribution of mean and standard deviation from Table S4). During high lion abundance, cubs' survival was the lowest and the values were sampled on the left hand side of the distribution (smallest 10% on the normal distribution of mean and standard deviation from Table S4). If lion abundance was average, cubs' survival was sampled in between the low and high ranges. Because the exact relationship between cub mortality and lion abundance is unknown, we undertook a scenario-based approach where various estimates of the impact of lion abundance on cheetah cub survival were considered. We varied the cut-off values defining the impact of lions on cub survival from 1% to 40%. We used the 10% cut-off for high and low lion abundance as this value led to the best r-squared value between observed and simulated cheetah abundance (Figure S1).

According to [43], between 1966 and 2003, the Serengeti lions have been exposed to CDV four times, with only one event leading to a lethal outbreak (1994). Since the vaccination programme started, there has been no lethal outbreak in the lion population. Before 1996, there thus appears to have been one lethal outbreak in 30 years [36], [44]. In order to predict cheetah population trends with regard to lion abundance, we wanted to measure the cheetah population growth rate λ with different lethal outbreak rates (Table S2). We expected that if the CDV was eradicated from the Serengeti, the lethal outbreak rate would be 0 over any timeframe. However, without vaccination the lethal outbreak rate could stay the same, decrease or increase. By taking a rate of 2 lethal outbreaks per 60 years, the assumption is that, over time, the risk of lethal outbreak without vaccination remained the same as before 1996, when vaccination started. We ran simulations, projecting the cheetah population over a 60-year timeframe under different CDV lethal outbreak rate scenarios, ranging from no possibility of lethal outbreaks, i.e., complete eradication through vaccination, and a rate of 6 lethal outbreaks, i.e., three times the observed rate without vaccination.

## Results

Both the cheetah IBM and the lion model were able to mimic observed abundances (Table 2). The r-squared values between model-generated and observed cheetah abundances were at least two times higher when the model was run with lion impact varying over time (Table 2 (c) and (d)) than when run while considering the impact of lions on the cheetah population to be constant (Table 2 (b)).

**Table 2:** R-squared values of models.

Model(s)	r^2^±s.d.	Correlation with data in
(a) Lion matrix model	0.46±0.18	[32]
(b) Cheetah IBM, no lion	0.10±0.06	Figure 1
(c) Cheetah IBM coupled with lion model	0.35±0.13	Figure 1
(d) Cheetah IBM coupled with published lion abundance	0.45±0.09	Figure 1

The lion matrix model (a) performance is assessed against published lion abundance from 1975 to 2003 [31]. The cheetah IBM performance is assessed against unpublished cheetah monitoring data from 1991 to 2010 (b and c) and from 1991–2003 for (d) as lion published abundance stops in 2003. Cheetah modelled abundance is compared to data from 1991, as opposed to data from 1982, because the latest estimates are the most reliable. s.d stands for standard deviation.

**Figure 2:**
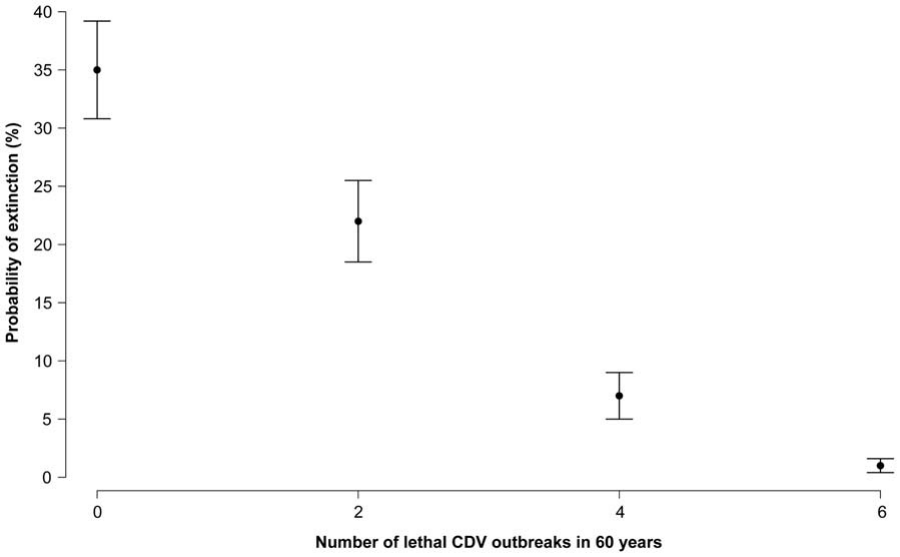
Probability of extinction of the Serengeti plains cheetah population for different rates of CDV outbreaks. Shown is the percentage of simulations iterations for which the population went extinct in 60 years (500 iterations were run for each outbreak rate). We assume that a rate of outbreak of 0 represents disease control through vaccination and a rate of 2 CDV outbreaks per 60 years is the rate without vaccination. Also shown is the probability of extinction of the population if the rate of CDV increases. The error bars represent the standard deviation.

Cheetah population projections showed that the chance of the population going extinct in the next 60 years drops from 35±4.2% when modelled under the vaccination scenario, i.e., no lethal outbreaks, to just over 20±3.5% under the 2 lethal outbreaks in 60 years scenario, i.e., no vaccination (Figure 2). We tested the sensitivity of this result to changes in initial cheetah population size by running the model with half and twice the actual initial population size. We found some variation in the amount by which the population's chances of going extinct increase with vaccination effort (Table S5). However, the conclusion that a lower CDV lethal outbreak rate negatively impacts the cheetah population in the long term was robust to changes in cheetah population size.

By simply doubling the number of outbreaks from 2 to 4 in 60 years, we found that the probability of the cheetah population going extinct was five times less than under the scenario where the outbreak rate is null (Figure 2). Simulations of the lion under the same outbreak rates, 2 and 4 in 60 years yielded a probability of extinction of 0 in both cases. Moreover, the lion population growth rate was λ = 1.0102±0.005 for a rate of 4 lethal outbreaks in 60 years. Our model suggested that a greater outbreak rate would not lead the lion population to extinction because the impact of a lethal CDV outbreak manifests itself through a sudden but temporary drop in lion numbers. It would, however greatly benefit the cheetah's chance of survival as a lower lion population size allows the cheetah population to augment due to an increase in cub survival. In addition, regardless of the number of outbreaks in 60 years explored here, the cheetah population was, at best, stable over this period. The average cheetah population growth rate for the highest lethal outbreak rate considered (6 in 60 years) was indeed λ = 1.001±0 017. This showed that for the cheetah population to be self-replacing, the lethal outbreak rate needed to be much higher than current rates.

## Discussion

Although vaccination programmes targeted towards wildlife conservation have become more widespread in the last decades [5], [45]–[52], the impact of using vaccination as a conservation tool on wildlife communities has rarely been investigated. Our work aimed to address this current gap and used the unique opportunity provided by the long-term monitoring of two long-lived populations of carnivores, to quantitatively assess how a vaccination programme targeting a single species can inadvertently put another threatened species under pressure. Our goal is not to discredit vaccination campaigns as a conservation tool since they can play an important conservation role, or to criticize a particular project. Instead our aim is to draw attention to the issue of unintended consequences of conservation actions, a problem recently highlighted by Harihar and co-authors, who reported that conservation measures to promote the survival of tigers (*Panthera tigris*) in India led to a sharp decline in the population of leopards (*Panthera pardus*) [53].

Based on previous studies, we suspected that lion density is likely to negatively impact cheetah population dynamics. Such an expectation was based on knowing that (1) cheetah biomass is inversely correlated with lion biomass across protected areas in the African sub-Sahara [54]; (2) cheetah cub mortality between birth and independence averages 95%, of which 78.2% has been shown to be due to predation by lions [30]; (3) cheetahs are more likely to be seen from scans with intermediate numbers of gazelles and where predators are absent while lions are more likely to be seen from scans with the highest number of gazelles [26]; (4) cheetahs are less likely to hunt in the vicinity of lions [55]; (5) annual reproductive success of female cheetahs correlates with their level of lion avoidance [56]; (6) cheetah recruitment is negatively correlated with the number of lions [28]. In addition, abundance records suggest that not only have cheetahs been decreasing since 1982, but, since 1996 (when the vaccination campaign started) the slope of the decrease has doubled (from 0.7 to 1.4, p<0.05).

Yet, to quantitatively assess the impact of a conservation action aiming at reducing CDV lethal outbreaks in lions on cheetah population viability, we needed to demonstrate that a cheetah model incorporating temporal information on lion density performed better than (1) a model with constant lion density or (2) a model without information on lion density. Thanks to our approach, we were not only able to reconstruct 45±9% of the variability in cheetah population size, but we were also able to demonstrate for the first time that accounting for lion density variation over time is key to cheetah population modelling. Considering our results and all the knowledge accumulated so far on lion-cheetah interactions in this system [26], [28], [30], [54]–[56] we believe more research should be carried out to parameterise such link.

Our results, however, do have some limitations as some of the links and assumptions incorporated in the models have not been empirically quantified. Several areas need further investigation: for example, (1) more research should be carried out to parameterise the relationship between lion density and cheetah cub survival, as this could increase our ability to predict cheetah population size in the SNP; (2) the true impact of the vaccination program on the probability of a lethal CDV outbreak occurring has not yet been assessed, and such information would immensely improve our model's predictive ability; (3) the mechanisms that trigger a lethal CDV outbreak are not clear and the relationship between CDV, lion density and lion mortality rates should be investigated further; (4) estimates of lion abundance are only available up until 2003 and more recent abundance estimates could improve the robustness of our conclusions.

The main assumption of our work is that CDV outbreaks are beneficial to lion and cheetah coexistence. Yet one could object that (1) CDV is not a regulatory mechanism of the lion population and thus not responsible for lion losses, and (2) CDV has only been recently introduced in the ecosystem. There is currently little support to the first objection, with the most parsimonious explanation proposed to link CDV and lion population dynamics involving a scenario where the lion population protection against CDV is due to stochasticity in pathogen circulation until 1981, then latency over a long period without any infection, resulting in the 1994 lethal outbreak [57]. A recent paper by Craft and colleagues did propose an alternative whereby the lethal CDV outbreak of 1994 was made possible by the high proximity of lion prides to each other and to other CDV-carrying carnivores [58], thus linking density to the probability of a lethal outbreak occurring. In both cases, however, lethal CDV outbreak is explicitly linked to past lion population losses.

As for the second objection, the timeframe of CDV introduction is difficult to assess: the Serengeti lion population survey began in 1966, and no case of disease was reported before 1994 [17]. We know that some lions born before 1981 presented CDV-antibodies, but no information is available about the presence or absence of CDV before 1981 [57]. Considering that CDV has been known to affect dogs for decades (the virus was first described in 1905; [59]), and considering that the use of herd dogs by pastoralists is traditional in these ecosystems [60], it is not unreasonable to support the alternative hypothesis, namely that CDV has not been recently introduced in the ecosystem.

The consideration of whether the CDV is a natural component of the ecosystem is, nonetheless, very important. As the role of CDV moves along the natural-human continuum, i.e., from a natural component of a functioning ecosystem towards a human-driven threat, the justification for a vaccination program becomes stronger. In such circumstances CDV could be compared to, for example, a threat posed by introduced species. Whether CDV is natural or not thus fundamentally changes approaches to managing the disease, since management of ecosystems is usually targeted at managing human-driven threats whilst retaining natural functioning components of ecosystems [61].

Another potential issue relating to this work is the assumption that CDV vaccinations in domestic dogs are responsible for the recent increase in lion numbers. It could be argued that such a vaccination programme hasn't decreased the rate of CDV exposure in lions, meaning that (1) the campaign has been/is inefficient at reducing outbreak risks, and (2) the vaccination campaign has not influenced (and won't influence) lion population dynamics. Based on the available literature and released information, it is difficult to assess the extent to which the campaign has been successful at reducing CDV exposure in lions and risks of high population losses. However, lion abundance records [32] show that before vaccination started, the population was neither increasing nor decreasing, but since 1996 it has been significantly increasing (slope = 11.7, p<0.05; Figure 1). One caveat is that published abundance records, and thus analysis, stop in 2003. Nonetheless, we have no reason to believe that lion numbers have not been at least stable since then. In order to help increase the accuracy and reliability of our quantitative assessment, further information should be sought and disseminated. Importantly, our work aims to highlight the potential unintended negative consequences of such a campaign on cheetah population dynamics and illustrate the importance of an ecosystem approach to disease management, where interactions among species are considered when implementing a conservation action such as a vaccination campaign.

Altogether, our results illustrate that the long-term impact of protecting one threatened species from disease could lead to unforeseen negative impacts on another threatened species. They also demonstrate how long-term targeted monitoring is key to identifying such impacts. In this system, monitoring should be continuously used in the future to further understand the effect of the vaccination programme on cheetah population viability and improve the robustness of our predictions. The cheetah population at present is not isolated, and can be maintained by population supplementation on its borders [27]. However, results from these simulations have clear implications for smaller and more isolated populations of cheetah outside of the Serengeti. Just as human intervention can disturb the trophic cascade by removing species, e.g., releasing meso-predators and increasing pressure on herbivores, human-promoted increase in a species' survival can also perturb trophic interactions. With this case study, we illustrate how human intervention can lead to unintentional conservation triage [59], that is, prioritising one species over the other could unknowingly lead to the decrease and even potential disappearance of a non-target species. It is now widely accepted that with current limits to funding, conservationists may be faced with a lose-lose situation where the options may be to (1) do nothing and potentially lose one or several species, (2) guarantee the safety of one or a few species but also condemn others to extinction [62]. It is, however, important to ensure that species do not become threatened with extinction as an inadvertent by-product of conservation interventions. In order to help ensure this is avoided, conservation planning should take an ecosystem approach, and ensure the impacts of conservation interventions on non-target species, particularly threatened species, are monitored and minimised.

## Supporting Information

Figure S1 **R-squared value between observed and modelled cheetah abundance depending on the association between lion density and cheetah cub survival.**
(TIF)

Table S1 **Population projection matrix for the Serengeti lions.** (a) for years during which there is no CDV outbreak, and (b) during which there is a CDV outbreak. The reproduction rate *F_x_* is randomly selected on a normal distribution of mean 0.65 and standard deviation of 0.11 in the following way: *F_x_* is taken from the left-hand side of the distribution (min to mean) if the population is over the carrying capacity and on the right-hand side (mean to max) of the distribution if the population is below carrying capacity.
(DOC)

Table S2 **Corresponding CDV outbreaks trigger numbers and CDV outbreak rates.**
(DOC)

Table S3 **Initial cheetah population size**.
(DOC)

Table S4 **Age- and sex- specific cheetah monthly survival rates.** s.d. stands for standard deviation.
(DOC)

Table S5 **Analysis of sensitivity to changes in initial cheetah population size.** The actual population size is the one presented in Supplementary table 4. ‘1/2× pop size’ is obtained by dividing the actual population size by two for each age- and sex-class. ‘2× pop size’ is obtained by multiplying the actual population size by two for each age- and sex-class. Each simulation was run for 500 iterations.
(DOC)
