# Correction: Unintended Consequences of Conservation Actions: Managing Disease in Complex Ecosystems

**DOI:** 10.1371/annotation/cf1771ad-01ca-430e-bccc-5fe1708f8902

**Published:** 2012-01-24

**Authors:** Aliénor L. M. Chauvenet, Sarah M. Durant, Ray Hilborn, Nathalie Pettorelli

In this article, the authors acknowledged Dr Sarah Cleaveland and Dr Craig Packer as having provided detailed comments on earlier versions of the manuscript. Although the authors sent a revised version to Dr Cleaveland mentioning her name in the acknowledgments before submission to PLoS ONE and received no correspondence suggesting any objection, they accept that she did not formally agree to be included in the Acknowledgments.

Similarly, the authors did not seek Dr Packer's formal agreement for him to be listed in the Acknowledgements. As a result, they would like to issue this Correction in order to remove the reference to Drs. Cleaveland and Packer and revise the text under the Acknowledgments to:

"We thank TANAPA, TAWIRI and the Tanzania Commission for Science and Technology for providing permission to conduct the long-term study in the Serengeti. We would like to thank all who have contributed to the fieldwork of the Serengeti Cheetah Project. Numerous people and organisations in Tanzania and Kenya have provided much needed logistical support over the years, including B. Allen, O. Newman, A. Barrett, J. Driessen, J. Jackson, A. Geertsema, P. and L. White and the staff and management of Ndutu Safari Lodge, H. van Lawick and his team, fellow scientists at SWRC, G. and M. Russell and M. Borner and the staff at FZS. We thank John Harwood for initial discussions, as well as Tim Coulson and Dominique Pontier for providing detailed comments on earlier versions of this manuscript. The authors also gratefully acknowledge the many organisations that have funded the project, principally The Howard G. Buffett Foundation, Wildlife Conservation Society, Frankfurt Zoological Society (FZS), National Geographic Society, Leverhulme Trust, Royal Society UK, Times Christmas Appeal 1998, Messerli Foundation and People's Trust for Endangered Species." 

