# Supplementary figures and images for: Unintended Consequences of Conservation Actions: Managing Disease in Complex Ecosystems
Source: PLoS One. 2011 Dec 7;6(12):e28671. doi: 10.1371/journal.pone.0028671 (PMC3233597; doi:10.1371/journal.pone.0028671)

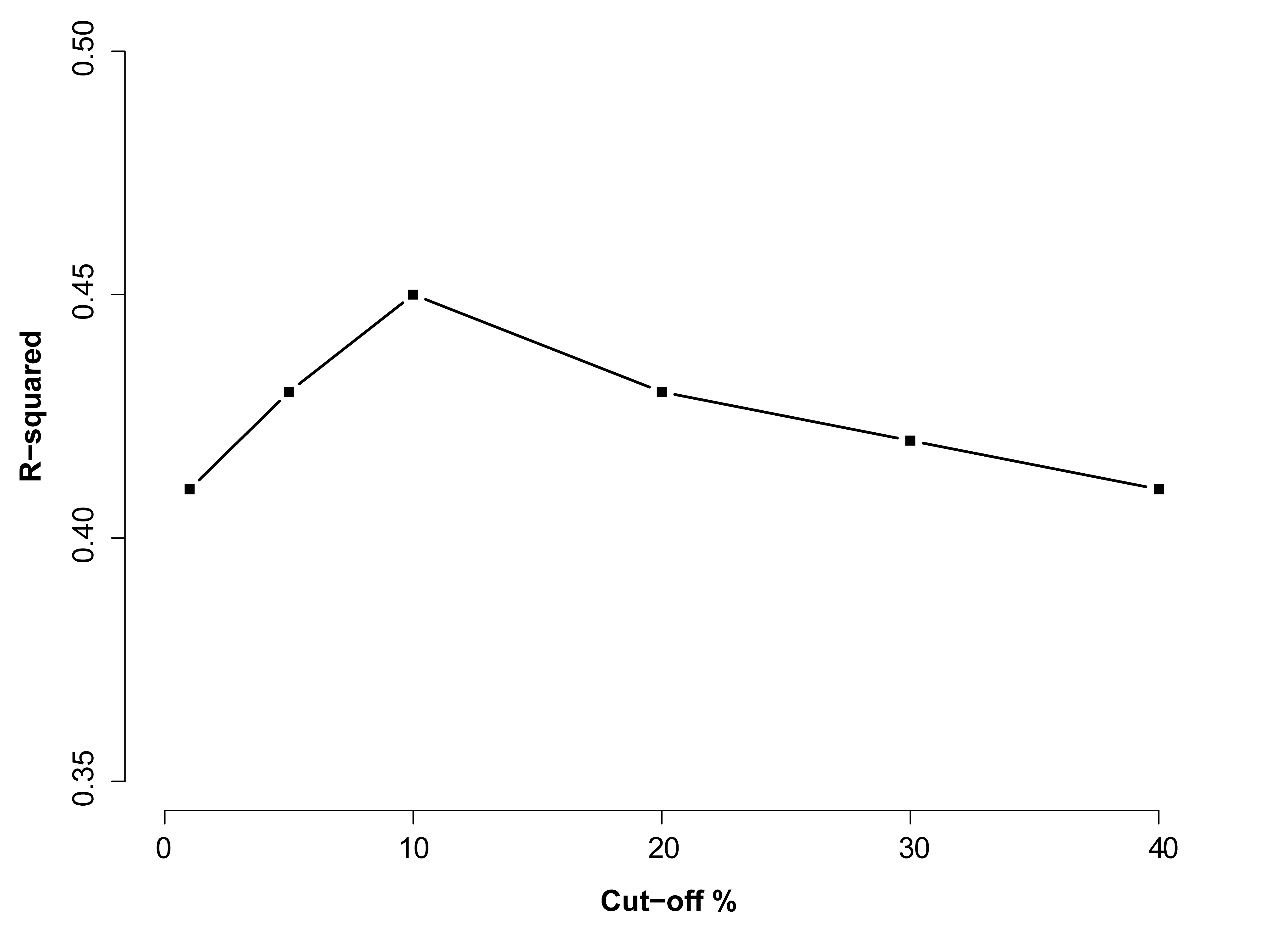

Supplement: Figure S1 — R-squared value between observed and modelled cheetah abundance depending on the association between lion density and cheetah cub survival. (TIF) [file pone.0028671.s001.tif]
