# Supplementary material for: Unintended Consequences of Conservation Actions: Managing Disease in Complex Ecosystems
Source: PLoS One. 2011 Dec 7;6(12):e28671. doi: 10.1371/journal.pone.0028671 (PMC3233597; doi:10.1371/journal.pone.0028671)
Supplement: Table S1 — Population projection matrix for the Serengeti lions. (a) for years during which there is no CDV outbreak, and (b) during which there is a CDV outbreak. The reproduction rate Fx is randomly selected on a normal distribution of mean 0.65 and standard deviation of 0.11 in the following way: Fx is taken from the left-hand side of the distribution (min to mean) if the population is over the carrying capacity and on the right-hand side (mean to max) of the distribution if the population is below carrying capacity. (DOC) [file pone.0028671.s002.doc]

**Table S1 a**.

| ***x*** | **0-1** | **1-2** | **2-3** | **3-4** | **4-5** | **5-6** | **6-7** | **7-8** | **8-9** | **9-10** | **10-11** | **11-12** | **12-13** | **13-14** | **14-15** | **15-16** | **16-17** | **17-18** |
| --- | --- | --- | --- | --- | --- | --- | --- | --- | --- | --- | --- | --- | --- | --- | --- | --- | --- | --- |
| **0-1** | 0.0 | 0.0 | 0.0 | ***F_3-4_*** | ***F_4-5_*** | ***F_5-6_*** | ***F_6-7_*** | ***F_7-8_*** | ***F_8-9_*** | ***F_9-10_*** | ***F_10-11_*** | ***F_11-12_*** | ***F_12-13_*** | ***F_13-14_*** | 0.0 | 0.0 | 0.0 | 0.0 |
| **1-2** | **0.4** | 0.0 | 0.0 | 0.0 | 0.0 | 0.0 | 0.0 | 0.0 | 0.0 | 0.0 | 0.0 | 0.0 | 0.0 | 0.0 | 0.0 | 0.0 | 0.0 | 0.0 |
| **2-3** | 0.0 | **0.9** | 0.0 | 0.0 | 0.0 | 0.0 | 0.0 | 0.0 | 0.0 | 0.0 | 0.0 | 0.0 | 0.0 | 0.0 | 0.0 | 0.0 | 0.0 | 0.0 |
| **3-4** | 0.0 | 0.0 | **0.9** | 0.0 | 0.0 | 0.0 | 0.0 | 0.0 | 0.0 | 0.0 | 0.0 | 0.0 | 0.0 | 0.0 | 0.0 | 0.0 | 0.0 | 0.0 |
| **4-5** | 0.0 | 0.0 | 0.0 | **0.9** | 0.0 | 0.0 | 0.0 | 0.0 | 0.0 | 0.0 | 0.0 | 0.0 | 0.0 | 0.0 | 0.0 | 0.0 | 0.0 | 0.0 |
| **5-6** | 0.0 | 0.0 | 0.0 | 0.0 | **0.9** | 0.0 | 0.0 | 0.0 | 0.0 | 0.0 | 0.0 | 0.0 | 0.0 | 0.0 | 0.0 | 0.0 | 0.0 | 0.0 |
| **6-7** | 0.0 | 0.0 | 0.0 | 0.0 | 0.0 | **0.9** | 0.0 | 0.0 | 0.0 | 0.0 | 0.0 | 0.0 | 0.0 | 0.0 | 0.0 | 0.0 | 0.0 | 0.0 |
| **7-8** | 0.0 | 0.0 | 0.0 | 0.0 | 0.0 | 0.0 | **0.9** | 0.0 | 0.0 | 0.0 | 0.0 | 0.0 | 0.0 | 0.0 | 0.0 | 0.0 | 0.0 | 0.0 |
| **8-9** | 0.0 | 0.0 | 0.0 | 0.0 | 0.0 | 0.0 | 0.0 | **0.9** | 0.0 | 0.0 | 0.0 | 0.0 | 0.0 | 0.0 | 0.0 | 0.0 | 0.0 | 0.0 |
| **9-10** | 0.0 | 0.0 | 0.0 | 0.0 | 0.0 | 0.0 | 0.0 | 0.0 | **0.9** | 0.0 | 0.0 | 0.0 | 0.0 | 0.0 | 0.0 | 0.0 | 0.0 | 0.0 |
| **10-11** | 0.0 | 0.0 | 0.0 | 0.0 | 0.0 | 0.0 | 0.0 | 0.0 | 0.0 | **0.9** | 0.0 | 0.0 | 0.0 | 0.0 | 0.0 | 0.0 | 0.0 | 0.0 |
| **11-12** | 0.0 | 0.0 | 0.0 | 0.0 | 0.0 | 0.0 | 0.0 | 0.0 | 0.0 | 0.0 | **0.9** | 0.0 | 0.0 | 0.0 | 0.0 | 0.0 | 0.0 | 0.0 |
| **12-13** | 0.0 | 0.0 | 0.0 | 0.0 | 0.0 | 0.0 | 0.0 | 0.0 | 0.0 | 0.0 | 0.0 | **0.9** | 0.0 | 0.0 | 0.0 | 0.0 | 0.0 | 0.0 |
| **13-14** | 0.0 | 0.0 | 0.0 | 0.0 | 0.0 | 0.0 | 0.0 | 0.0 | 0.0 | 0.0 | 0.0 | 0.0 | **0.7** | 0.0 | 0.0 | 0.0 | 0.0 | 0.0 |
| **14-15** | 0.0 | 0.0 | 0.0 | 0.0 | 0.0 | 0.0 | 0.0 | 0.0 | 0.0 | 0.0 | 0.0 | 0.0 | 0.0 | **0.7** | 0.0 | 0.0 | 0.0 | 0.0 |
| **15-16** | 0.0 | 0.0 | 0.0 | 0.0 | 0.0 | 0.0 | 0.0 | 0.0 | 0.0 | 0.0 | 0.0 | 0.0 | 0.0 | 0.0 | **0.6** | 0.0 | 0.0 | 0.0 |
| **16-17** | 0.0 | 0.0 | 0.0 | 0.0 | 0.0 | 0.0 | 0.0 | 0.0 | 0.0 | 0.0 | 0.0 | 0.0 | 0.0 | 0.0 | 0.0 | **0.4** | 0.0 | 0.0 |
| **17-18** | 0.0 | 0.0 | 0.0 | 0.0 | 0.0 | 0.0 | 0.0 | 0.0 | 0.0 | 0.0 | 0.0 | 0.0 | 0.0 | 0.0 | 0.0 | 0.0 | **0.1** | 0.0 |

**Table S1 b.**

| ***x*** | **0-1** | **1-2** | **2-3** | **3-4** | **4-5** | **5-6** | **6-7** | **7-8** | **8-9** | **9-10** | **10-11** | **11-12** | **12-13** | **13-14** | **14-15** | **15-16** | **16-17** | **17-18** |
| --- | --- | --- | --- | --- | --- | --- | --- | --- | --- | --- | --- | --- | --- | --- | --- | --- | --- | --- |
| **0-1** | 0.0 | 0.0 | 0.0 | ***F_3-4_*** | ***F_4-5_*** | ***F_5-6_*** | ***F_6-7_*** | ***F_7-8_*** | ***F_8-9_*** | ***F_9-10_*** | ***F_10-11_*** | ***F_11-12_*** | ***F_12-13_*** | ***F_13-14_*** | 0.0 | 0.0 | 0.0 | 0.0 |
| **1-2** | **0.3** | 0.0 | 0.0 | 0.0 | 0.0 | 0.0 | 0.0 | 0.0 | 0.0 | 0.0 | 0.0 | 0.0 | 0.0 | 0.0 | 0.0 | 0.0 | 0.0 | 0.0 |
| **2-3** | 0.0 | **0.6** | 0.0 | 0.0 | 0.0 | 0.0 | 0.0 | 0.0 | 0.0 | 0.0 | 0.0 | 0.0 | 0.0 | 0.0 | 0.0 | 0.0 | 0.0 | 0.0 |
| **3-4** | 0.0 | 0.0 | **0.7** | 0.0 | 0.0 | 0.0 | 0.0 | 0.0 | 0.0 | 0.0 | 0.0 | 0.0 | 0.0 | 0.0 | 0.0 | 0.0 | 0.0 | 0.0 |
| **4-5** | 0.0 | 0.0 | 0.0 | **0.8** | 0.0 | 0.0 | 0.0 | 0.0 | 0.0 | 0.0 | 0.0 | 0.0 | 0.0 | 0.0 | 0.0 | 0.0 | 0.0 | 0.0 |
| **5-6** | 0.0 | 0.0 | 0.0 | 0.0 | **0.9** | 0.0 | 0.0 | 0.0 | 0.0 | 0.0 | 0.0 | 0.0 | 0.0 | 0.0 | 0.0 | 0.0 | 0.0 | 0.0 |
| **6-7** | 0.0 | 0.0 | 0.0 | 0.0 | 0.0 | **0.7** | 0.0 | 0.0 | 0.0 | 0.0 | 0.0 | 0.0 | 0.0 | 0.0 | 0.0 | 0.0 | 0.0 | 0.0 |
| **7-8** | 0.0 | 0.0 | 0.0 | 0.0 | 0.0 | 0.0 | **0.7** | 0.0 | 0.0 | 0.0 | 0.0 | 0.0 | 0.0 | 0.0 | 0.0 | 0.0 | 0.0 | 0.0 |
| **8-9** | 0.0 | 0.0 | 0.0 | 0.0 | 0.0 | 0.0 | 0.0 | **0.7** | 0.0 | 0.0 | 0.0 | 0.0 | 0.0 | 0.0 | 0.0 | 0.0 | 0.0 | 0.0 |
| **9-10** | 0.0 | 0.0 | 0.0 | 0.0 | 0.0 | 0.0 | 0.0 | 0.0 | **0.7** | 0.0 | 0.0 | 0.0 | 0.0 | 0.0 | 0.0 | 0.0 | 0.0 | 0.0 |
| **10-11** | 0.0 | 0.0 | 0.0 | 0.0 | 0.0 | 0.0 | 0.0 | 0.0 | 0.0 | **0.7** | 0.0 | 0.0 | 0.0 | 0.0 | 0.0 | 0.0 | 0.0 | 0.0 |
| **11-12** | 0.0 | 0.0 | 0.0 | 0.0 | 0.0 | 0.0 | 0.0 | 0.0 | 0.0 | 0.0 | **0.5** | 0.0 | 0.0 | 0.0 | 0.0 | 0.0 | 0.0 | 0.0 |
| **12-13** | 0.0 | 0.0 | 0.0 | 0.0 | 0.0 | 0.0 | 0.0 | 0.0 | 0.0 | 0.0 | 0.0 | **0.5** | 0.0 | 0.0 | 0.0 | 0.0 | 0.0 | 0.0 |
| **13-14** | 0.0 | 0.0 | 0.0 | 0.0 | 0.0 | 0.0 | 0.0 | 0.0 | 0.0 | 0.0 | 0.0 | 0.0 | **0.5** | 0.0 | 0.0 | 0.0 | 0.0 | 0.0 |
| **14-15** | 0.0 | 0.0 | 0.0 | 0.0 | 0.0 | 0.0 | 0.0 | 0.0 | 0.0 | 0.0 | 0.0 | 0.0 | 0.0 | **0.5** | 0.0 | 0.0 | 0.0 | 0.0 |
| **15-16** | 0.0 | 0.0 | 0.0 | 0.0 | 0.0 | 0.0 | 0.0 | 0.0 | 0.0 | 0.0 | 0.0 | 0.0 | 0.0 | 0.0 | **0.3** | 0.0 | 0.0 | 0.0 |
| **16-17** | 0.0 | 0.0 | 0.0 | 0.0 | 0.0 | 0.0 | 0.0 | 0.0 | 0.0 | 0.0 | 0.0 | 0.0 | 0.0 | 0.0 | 0.0 | **0.0** | 0.0 | 0.0 |
| **17-18** | 0.0 | 0.0 | 0.0 | 0.0 | 0.0 | 0.0 | 0.0 | 0.0 | 0.0 | 0.0 | 0.0 | 0.0 | 0.0 | 0.0 | 0.0 | 0.0 | **0.0** | 0.0 |
