# Supplementary material for: Unintended Consequences of Conservation Actions: Managing Disease in Complex Ecosystems
Source: PLoS One. 2011 Dec 7;6(12):e28671. doi: 10.1371/journal.pone.0028671 (PMC3233597; doi:10.1371/journal.pone.0028671)
Supplement: Table S2 — Corresponding CDV outbreaks trigger numbers and CDV outbreak rates. (DOC) [file pone.0028671.s003.doc]

**Table S2.**

| **CDV outbreak number for a period of 60 years** | **Trigger number** |
| --- | --- |
| 0 (no CDV outbreak; vaccination) | 1 |
| 2 (no vaccination) | 0.95 |
| 4 | 0.82 |
| 6 | 0.3 |
