# Supplementary material for: Unintended Consequences of Conservation Actions: Managing Disease in Complex Ecosystems
Source: PLoS One. 2011 Dec 7;6(12):e28671. doi: 10.1371/journal.pone.0028671 (PMC3233597; doi:10.1371/journal.pone.0028671)
Supplement: Table S3 — Initial cheetah population size. (DOC) [file pone.0028671.s004.doc]

**Table S3**.

| **Age group (in months)** | **Males** | **Females** |
| --- | --- | --- |
| **0-12** | 30 | 30 |
| **13-24** | 4 | 5 |
| **25+ (reproducing class)** | 17 | 37 |
