# Supplementary material for: Unintended Consequences of Conservation Actions: Managing Disease in Complex Ecosystems
Source: PLoS One. 2011 Dec 7;6(12):e28671. doi: 10.1371/journal.pone.0028671 (PMC3233597; doi:10.1371/journal.pone.0028671)
Supplement: Table S4 — Age- and sex- specific cheetah monthly survival rates. s.d. stands for standard deviation. (DOC) [file pone.0028671.s005.doc]

**Table S4.**

| **Age group (in months)** | **Females** | | **Males** | | **Source** |
| --- | --- | --- | --- | --- | --- |
|  | **mean** | **s.d.** | **mean** | **s.d.** |  |
| **0-12** | 0.955 | 0.011 | 0.955 | 0.011 | [42] |
| **13-24** | 0.965 | 0.011 | 0.94 | 0.011 | [27,28] |
| **25+** | 0.987 | 0.0011 | 0.97 | 0.0011 | [27,28] |
