# Supplementary material for: Unintended Consequences of Conservation Actions: Managing Disease in Complex Ecosystems
Source: PLoS One. 2011 Dec 7;6(12):e28671. doi: 10.1371/journal.pone.0028671 (PMC3233597; doi:10.1371/journal.pone.0028671)
Supplement: Table S5 — Analysis of sensitivity to changes in initial cheetah population size. The actual population size is the one presented in Supplementary table 4. ‘1/2× pop size’ is obtained by dividing the actual population size by two for each age- and sex-class. ‘2× pop size’ is obtained by multiplying the actual population size by two for each age- and sex-class. Each simulation was run for 500 iterations. (DOC) [file pone.0028671.s006.doc]

**Table S5.**

| **Sensitivity analysis** | **Initial Cheetah number** | | |
| --- | --- | --- | --- |
|  | **Actual pop size** | **1/2x pop size** | **2x pop size** |
| Number by which the *probability of the population going extinct within 60 years* increases when model run with 0 outbreaks in 60 years compared to when run with 2 outbreaks in 60 years | 1.6 | 1.3 | 3 |
| Number by which the *average number of cheetahs still alive after 60 years* increases when model run with to 2 outbreaks in 60 years compared to when run with 0 outbreaks in 60 years | 2.1 | 1.8 | 2.2 |
